# The Impact of Zinc Oxide Nanoparticles on Cytotoxicity, Genotoxicity, and miRNA Expression in Barley (*Hordeum vulgare* L.) Seedlings

**DOI:** 10.1155/2020/6649746

**Published:** 2020-11-30

**Authors:** Ilona Plaksenkova, Inese Kokina, Anastasija Petrova, Marija Jermaļonoka, Vjačeslavs Gerbreders, Marina Krasovska

**Affiliations:** ^1^Department of Biotechnology, Institute of Life Sciences and Technology, Daugavpils University, Daugavpils 5401, Latvia; ^2^Department of Technology, Institute of Life Sciences and Technology, Daugavpils University, Daugavpils 5401, Latvia

## Abstract

Zinc oxide nanoparticles are one of the most commonly engineered nanomaterials and necessarily enter the environment because of the large quantities produced and their widespread application. Understanding the impacts of nanoparticles on plant growth and development is crucial for the assessment of probable environmental risks to food safety and human health, because plants are a fundamental living component of the ecosystem and the most important source in the human food chain. The objective of this study was to examine the impact of different concentrations of zinc oxide nanoparticles on barley *Hordeum vulgare* L. seed germination, seedling morphology, root cell viability, stress level, genotoxicity, and expression of miRNAs. The results demonstrate that zinc oxide nanoparticles enhance barley seed germination, shoot/root elongation, and H_2_O_2_ stress level and decrease root cell viability and genomic template stability and up- and downregulated miRNAs in barley seedlings.

## 1. Introduction

Nanotechnology and engineered nanoparticles (NPs) have received significant attention worldwide in recent years. Nanomaterials and nanoparticles have novel physical, chemical, mechanical, optical, and biological properties due to their large surface-to-volume ratio [1, 2]. This ratio expands the potential applications of nanomaterials in a wide range of industries and products, such as medicine, biosensors, cosmetics, food, the automotive industry, and clothing [3–5]. Advances in nanotechnology and the use of nanomaterials of different sizes, shapes, types, and chemical compositions are causing the accumulation of NPs in the environment. Therefore, worldwide attention is increasingly devoted to the issues of nano-biosecurity and the impact of NPs on living organisms and the environment as a whole [6, 7].

Plants are a fundamental component of ecosystem, as they are main producers and a significant part of the food chain [5, 8, 9]. Plants are very susceptible to nanotoxicity because they can absorb and accumulate NPs from soil, water, and air. Therefore, plants as a model organism are recommended as a first-level bioassay system to define the possible toxicity of various nanomaterials [10, 11]. Barley (*Hordeum vulgare* L.) is the fourth most important cereal crop worldwide; as such, it is one of the major crops grown for human and animal consumption [12, 13]. Because of its diploid nature and rich genetic diversity, barley is successfully used as a model organism for genetic studies [14, 15]. However, a limited number of studies on the effects of NPs on barley after a short exposure time have been conducted [16].

Stress caused by biotic and abiotic factors and the associated long-term use of fungicides, insecticides, and other chemical compounds sooner or later lead to reduced crop yield and quality [17, 18]. Therefore, a great deal of attention is devoted to the promotion of suitable agrotechnology activities using different nanobiotechnology methods to ensure sustainable agriculture [19–22]. The use of nanobiotechnology provides a practical solution to many agricultural problems, such as helping to improve the farming industry by increasing the efficiency of raw materials and reducing the corresponding losses. Moreover, the nanoparticles can be effectively used as an agrochemical carrier for controlled nutrient transport to increase yield [23–25].

Various NPs can induce stress responses in plants, affecting morphological, physiological, molecular, and biochemical reactions in the plant [26–28]. Previous studies have found that NPs can cause phytotoxicity, cytotoxicity, genotoxicity, or oxidative stress in plants, depending on the plant size, plant type, NP concentration, exposure time, and plant species [4, 29–31]. Zinc oxide (ZnO) NPs are one of the most produced nanomaterials worldwide (on a mass basis). These NPs are used in several industrial products, such as sunscreens, cosmetics, and paints [3, 32]. Moreover, ZnO NPs have been proposed as a fertiliser to supply Zn to plants [33].

Mildew is a plant disease caused by obligatory biotrophic fungi (*Ascomycota phylum*) [34]. Every year, plant disease leads to large losses in agricultural yields. Disease control involves the use of fungicidal and/or resistant plant varieties. Frequent prophylactic use of fungicides leads to the release of fungicides into the aquatic environment, which spread to surface waters throughout the growing season. Many studies have affirmed that fungicides can be very toxic to all living organisms [17, 18, 35–37]. The sustainable solution to this problem may be the use of nanoparticles as an effective tool for plant resistance-related microRNA (miRNA) regulation.

MicroRNAs are small endogenous, single-stranded noncoding RNA sequences. They are 20–24 nucleotides in length and are detected in all eukaryotic organisms [38]. MicroRNA plays a pivotal role in the regulation of posttranscriptional gene expression; it binds complementarily to the target messenger RNA (mRNA) and cleaves it or inhibits its translation process, thereby inhibiting gene expression [39–42]. Plant miRNA molecules are characterised by a variety of biological functions that are involved in the regulation of growth and development as well as the response to environmental stressors. Some plant miRNAs can regulate target gene expression, thereby helping the plant to survive in a changing environment. Therefore, miRNA-based technology is a highly efficient, reliable, and feasible technique for developing plant lines with increased stress tolerance [43–46]. The miRNAs miR156 and miR159 are intensively studied because they are involved in various plant responses to stress, such as responses to drought, hypoxia, fungal infections, and NPs [47, 48]. In wheat, miR159 is involved in response to fungal infection [49]. Nevertheless, the expression of miRNAs under exposure to ZnO NPs has not been analysed in a widely grown crop such as barley *H. vulgare* L. Therefore, there is a need to assess the critical response of barley seedlings to nanoscale ZnO materials (ZnO NPs) to understand the underlying mechanisms associated with ZnO NPs stress response.

To determine the impact of ZnO NPs on barley *Hordeum vulgare* L., the following objectives were established in the present study: (I) to investigate the effect of ZnO NPs on barley seed germination and plant morphology and (II) to investigate the cytotoxic and genotoxic effects and stress level and evaluate the miRNA expression levels caused by ZnO NPs in barley seedlings grown on hydroponics.

## 2. Materials and Methods

### 2.1. Exposure Suspension

The stock solutions were prepared by first dissolving 0.1 M Zn(CH_3_COO)_2_ ∗ 2H_2_O (Sigma Aldrich, ≥98%) in 50 mL of ethanol with continuous stirring. Secondly, 25 mL of 0.2 M NaOH (Merck, ≥99%) dissolved in ethanol were added dropwise to the stock solution until a pH value of 11 was reached. The obtained solution was ultrasonically stirred for one hour. The solution was then poured into a sealed Teflon-lined beaker and placed for six hours in an oven preheated to 90°C. The white precipitate was collected, rinsed with distilled water, and dried in the oven at 90°C. The result was a white powder consisting of spherical NP agglomerates.

Prior to use, the white powder was diluted in water to the required concentrations (0 (control), 1, 2, and 4 mg/L) and sonicated for one hour to separate the formed NPS agglomerates into individual NPs.

### 2.2. Seed Germination and Shoot/Root Elongation

Barley (*Hordeum vulgare* L. var. Abava) seeds were chosen for this study because of their known susceptibility to *Blumeria graminis* f. sp. hordei (Bgh). The seeds were supplied by the Institute of Agricultural Resources and Economics at the Stende Research Center. The seeds (*n* = 140 per each group) were visually inspected for any morphological damage or discoloration, soaked in 2.5% NaClO for 15 minutes and then in ethanol for five minutes and finally washed several times with sterilised deionised water. The seeds were transferred into 15 mm Petri dishes containing 8 mL of deionised water (control) or 8 mL of ZnO suspension at different concentrations. The Petri dishes were placed in the dark at 21°C for seven days. The germination percentage was calculated as the ratio of germinated seeds to total seeds in each Petri dish. A second set of seeds was treated for 14 days in the same conditions to evaluate root/shoot elongation, the number of seminal roots, cytotoxicity, genotoxicity, and the amount of chlorophyll *a*, *b* and miRNA. The image processing program ImageJ (open-source software provided by the National Institute of Health (NIH), hyperlink: http://rsbweb.nih.gov/ij/download.html) was used to measure the length of roots and shoots. The experiments were performed in triplicate.

### 2.3. Oxidative Stress Measurement Using DCFH-DA Staining

On the tenth day after treatment, the H_2_O_2_ content was analysed in fresh barley leaf/root samples (*n* = 20 per each group) to measure the level of oxidative stress in the plants. The tissues were incubated for 15 minutes in the dark with 2′,7′-dichlorofluorescein diacetate. The H_2_O_2_-treated leaves/roots were used as a positive control. The natural fluorescence of the leaves/root was also measured. The H_2_O_2_ content was estimated from the difference in intensity between the dye-treated and untreated samples using a Nikon Eclipse 80i fluorescence microscope equipped with a 488 nm laser. The fluorescence intensity was quantified using the image processing and analysis software Image J.

### 2.4. Cytotoxicity Evaluation Using Evans Blue Dye

The loss of cell viability was investigated applying the Evans blue staining method. The control and treated roots (*n* = 20 per each group) were stained with a 0.25% (w/v) aqueous solution of Evans blue for 15 minutes and then washed in distilled water for 30 minutes. Heat-treated roots were utilized as a positive control, and water was used as a negative control for the experiment. Further, the roots were macroimaged for a qualitative evaluation of cell death. For a quantitative estimation, 10 root tips of equal length from each experimental group were cut off and soaked in 4 mL of N,N-dimethylformamide for one hour at room temperature. The absorbance of the dye released was measured at 600 nm using the same microscope.

### 2.5. Genotoxicity Evaluation

#### 2.5.1. Random Amplified Polymorphic DNA (RAPD) Analysis

The genotoxic effects induced by ZnO NPs were estimated through random amplified polymorphic DNA (RAPD) analysis. The obtained seedling leaves were used for DNA extraction (*n* = 50 per each group). The extraction of total genomic plant DNA was performed using the Mini protocol: purification of total DNA from plant tissue (DNeasy Plant Mini Kit, Qiagen GmbH, Germany) via the QIAcube (Qiagen, Germany) extraction system. The DNA was extracted from approximately 90 mg of wet plant leaves. The final elution volume of the DNA was 150 *μ*L. The DNA was quantified and qualified through the use of a spectrophotometer (NanoDrop 1000, Thermo Scientific, USA).

A total of five decamer primers were selected for RAPD analysis: CB-21, OPA-02, OPA-05, OPA-11, and OPD-18. The RAPD analysis and electrophoresis were performed based on the method reported by Plaksenkova et al. [48]. Polymerase chain reaction (PCR) amplification was performed using the tiTaq PCR Master Mix (2x) (EURx, Poland) according to the manufacturer's protocol, with slight modifications.

The PCR reaction products were electrophoresed through the use of the QIAxcel Advanced (Qiagen, Germany) instrument utilising the QIAxcel DNA high-resolution kit according to the protocol for determination of DNA fragment sizes under the QIAxcel ScreenGel Software (Qiagen, Germany). QX Size Marker 100 bp–2.5 kb and QX Alignment Marker 15 bp/3 kb (Qiagen, Germany) were utilized to determinate the DNA fragment sizes. The RAPD fragments were marked up for the presence or absence of band products for all tested primers. The amplification reaction for each primer was repeated twice for each sample to provide reproducibility. Only clear and reproducible bands were considered for analysis.

#### 2.5.2. Evaluation of Genomic Template Stability

Genomic template stability (GTS, %) was calculated using the equation reported by Salarizadeh and Kavousi [50]:

(1) $$\begin{array}{c} \text{GTS}\left(\%\right)=\left(\frac{1-a}{n}\right)\times 100, \end{array}$$

where *a* is the average number of changes in each experimental group DNA profile, and *n* is the number of total bands in the control samples [50]. The polymorphic bands observed in the RAPD analysis were defined as the gain or loss of bands in comparison with the control profile. The average number of polymorphic bands was calculated for each experimental group.

#### 2.5.3. MicroRNA Level Evaluation by RT-qPCR

Two-step qPCR analysis was performed to estimate the expression of miRNAs in barley plants grown under different concentrations of ZnO NPs and in control plants (*n* = 50 per each group). Ribonucleic acid extraction, first-strand cDNA synthesis, and real-time PCR were performed according to the method reported by Plaksenkova et al. [48].

MicroRNA target-specific primers lus-miR159c, hvu-miR159a, and hvu-miR156a with locked nucleic acids were designed. The miRNA sequences were as follows:

  lus-miR159c target sequence: 5′-UUUGGAUUGAAGGGAGCUCUU-3′
  hvu-miR159a input sequence: 5′-TTTGGATTGAAGGGAGCTCTG-3′
  hvu-miR156a input sequence: 5′-TGACAGAAGAGAGTGAGCACA-3′

In the relative quantification analysis, the elongation factor 1-alpha (EF1*α*) gene [51] was used as a reference gene to normalise the expression values.

### 2.6. Statistical Analysis

Three biological replicates were considered in each experiment and presented as mean ± standard deviation. Significant differences among the treatments were measured through *t*-tests. The level of significance was established at *p* < 0.05 and *p* < 0.01.

## 3. Results and Discussion

### 3.1. Particle Characterisation

**Figure 1:**
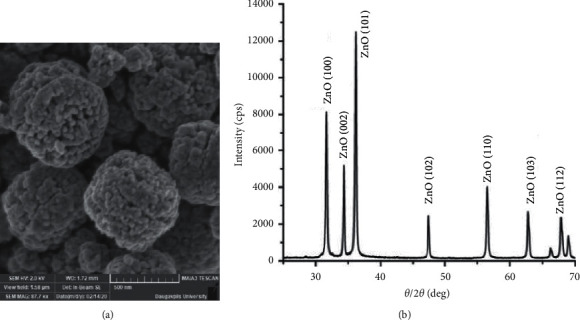
(a) SEM image of spherical agglomerates of ZnO nanostructures and (b) XRD pattern of ZnO nanostructured powder.

The size of individual NPs was determined using PDXL software according to the Williamson–Hall method, and the nanoparticle value was 31 nm. The surface morphology of the processed samples was examined using a scanning electron microscope (TESCAN Maia3). For the determination of the structural and phase composition, the XRD spectra were recorded by a SmartLab Cu K*α* (*λ* = 1.543 Å) diffractometer (Rigaku) with parallel beam geometry using an additional Ge(220) × 2 monochromator (Figure 1). The X-ray diffraction results revealed that the samples were crystalline with a hexagonal wurtzite structure corresponding to ZnO. No other phase inclusions were detected. In addition, the low levels of amorphous background reveal that the nanostructures had a high degree of crystallinity.

Zinc is an essential plant micronutrient and is often supplied as zinc sulfate in agricultural practice to overcome zinc deficiency in plants. Zinc acts as a cofactor for a number of metabolic and physiological cycles by influencing the activities of RNA polymerases and other plant enzymes. Zinc oxide NPs increase the activity of phosphorous mobilising enzymes, such as phosphatase and phytases in the rhizosphere, thereby increasing the amount of phosphorous available to plants [52, 53]. Thus, the enhanced physiological and biochemical response are consistent with the twin role of ZnO NPs as essential nutrients and mobilisers of native phosphorous [54]. Moreover, ZnO NPs exhibit biological compatibility and structural stability [55].

### 3.2. Germination and Root Elongation

The effects of ZnO NPs on seed germination and shoot and root growth are presented in Table 1. All tested concentrations of NPs resulted in a significant increase (*p* < 0.01) in the seed germination of barley (*H. vulgare* L.). The highest germination rate (66%) was observed for the seeds germinated with the addition of NPs at 4 mg/L, whereas the control seeds demonstrated a significantly lower germination rate, at only 42%. The germination rate at 1 mg/L and 2 mg/L was 63% and 57%, respectively. There was a significant effect of ZnO NPs (*p* < 0.05 and *p* < 0.01) on the average length of shoots. There was no statistically significant difference (*p* > 0.05) between the root lengths of seedlings and the number of seminal roots. In fact, the highest concentration of ZnO NPs (4 mg/L) most strongly affected barley germination and shoot and root length.

Seed germination and root/shoot elongation tests have been previously used to estimate short-term phytotoxicity for the assessment of the ecological risks posed by emerging pollutants, such as engineered NPs [56]. One previous study found that ZnO NPs at concentrations of 1–80 mg/L insignificantly affected the germination of Chinese cabbage seeds compared with the control [56]. In addition, Raliya et al. [54] reported that tomato seed germination was not affected by ZnO NPs in concentrations up to 750 mg/kg. Moreover, Zhang et al. [57] found no statistically significant reduction in the germination rate of corn and cucumber treated with ZnO NPs at concentrations of 10, 100, and 1,000 mg/L. However, the results of the present study indicate a significant increase in barley seed germination under low ZnO NP concentrations. Thus, the germination rate seems to be concentration- and species-dependent.

Zinc oxide NPs at 100, 500, and 1,000 mg/L concentrations reduced the root length and number of roots in germinated rice seedlings [58]. Moreover, ZnO NPs <30 ± 10 nm at a concentration of 1,000 g/L reduced the root length in maize compared to the control. However, ZnO NPs at a concentration of 10 mg/L significantly promoted the root length of germinated corn [57]. Exposure to ZnO NPs with sizes <50 nm and concentrations of 5, 10, 25, 50, 75, 100, 125, 250, and 500 *µ*g/mL decreased the shoot and root length of rapeseed germinated seedlings [59]. However, 20 nm ZnO NPs at concentrations of 10, 20, 30, and 40 mg/L increased onion seed germination at lower NP concentrations, but decreased germination at higher concentrations [60]. Furthermore, treatment of ZnO NPs at a concentration of 1,000 ppm was observed to significantly promote seed germination, but the higher concentration of ZnO NPs at 2,000 ppm was observed to have a negative and toxic effect on the growth and yield of peanuts [61].

Overall, ZnO NPs reduce or improve the seed germination of many plants. The plant response varies significantly among plant species and is partially correlated with the dose and size of the NPs [33]. The results of the present study can be explained by the fact that lower concentrations of ZnO NPs create a positive impact on shoot and root elongation in germinated barley seedlings. However, the mechanisms behind germination are still poorly understood.

### 3.3. Plant Growth and Biomass

**Figure 2:**
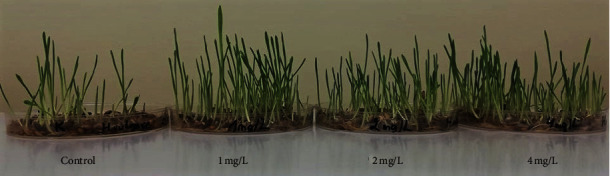
Germinated barley seedling growth under ZnO NPs stress conditions on the 7th day of exposure.

To investigate the impact of ZnO NPs on barley growth, the seedling growth parameters were recorded 14 days after exposure. The shoot and root height, shoot weights, and number of roots are presented in Table 2. The shoot and root length and number of roots significantly (*p* < 0.01) increased after treatment with different concentrations of ZnO NPs. However, shoot biomass was not significantly affected by ZnO NPs. The germinated barley seedling growth under different ZnO NPs stress conditions is presented in Figure 2. These results indicate that ZnO NPs are involved in promoting plant growth.

The same results were obtained by Mahajan et al. [62]. In their study, ZnO NPs (1∼20 ppm) increased the growth of mung bean and chickpea. Zinc oxide NPs at concentrations of 400 mg/kg and 800 mg/kg enhanced the growth of cucumber in soil [63]. Venkatachalam et al. [64] also reported that phytomolecules-loaded ZnO NPs at concentrations of 25 mg/L also enhanced *Leucaena leucocephala* seedling growth in hydroponic growth conditions. Concentrations of ZnO NPs in soil of up to 250 mg/kg significantly enhanced tomato seedling growth [54]. In addition, 34 nm large ZnO NPs (up to 100 mg/L) significantly increased wheat plant growth and biomass in soil [65]. However, ZnO NPs <100 nm (500 mg/kg) in size reduced the root and shoot length and biomass in wheat [66]. In addition, ZnO NPs <50 nm in size at 100 µg/mL exposure in hydroponics decreased rapeseed growth and biomass [67]. However, ZnO NPs <100 nm in size at concentrations of 200, 500, 1,000, and 1,500 *µ*g/mL decreased the shoot and root length and biomass of Indian mustard grown in hydroponic conditions [55]. Shoot and root length and biomass also decreased in onion seedlings upon exposure to 20 nm ZnO NPs at concentrations of 10, 20, 30, and 40 mg/L [60]. Furthermore, a reduction in root length as well as root weight was recorded in NP-stressed maize plants. However, no significant difference in the shoot length of control seedlings and those exposed to ZnO NPs <50 nm was observed [52]. Treatment of ZnO NPs at a concentration of 1,000 ppm was observed to significantly promote peanut seedling length and weight, but the higher concentration of ZnO NPS at 2,000 ppm was observed to have a negative and toxic effect on the growth and yield [61].

In short, ZnO NPs have both positive and negative effects on plant growth and morphology, and the effect varies with the dose applied, plant species, experimental conditions, and exposure duration. In *H. vulgare* L. seedlings, exposure to 32 nm ZnO NPs (1, 2, and 4 mg/L) in hydroponics increases the root and shoot length and germination rate.

### 3.4. Cytotoxicity

**Figure 3:**
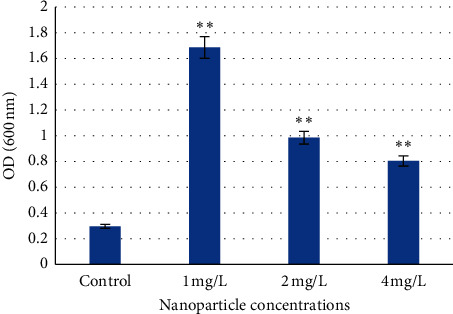
Evans blue dye exclusion assay: cytotoxicity of ZnO NPs in roots of *H. vulgare* L. seedlings. Values are the mean of three replicates with SD. ^*∗∗*^indicates significant difference from control (*p* < 0.01).

This study utilized Evans blue dye as a marker of membrane integrity to examine the cytotoxic effects of ZnO NPs in *H. vulgare* L. seedlings. Living cells are able to eliminate the dye at the plasma membrane, whereas cells with a damaged membrane are incapable of excluding the dye and are stained blue as a result [68]. This study found that ZnO NP treatment induced cell death in the root cells of barley seedlings. The roots exposed to NPs exhibited a higher uptake of dye compared to control roots. An approximate 3-5.5-fold increase in Evans blue uptake was observed at all concentrations tested. In addition, the smallest ZnO NPs concentrations demonstrated the highest fluorescence (mean chlorophyll fluorescence = 1.69 ± 0.92) compared to the control (mean chlorophyll fluorescence = 0.3 ± 0.59) (Figure 3). The obtained results indicate an effect of ZnO NPs on barley seedling cell viability and a significant (*p* < 0.01) increase in cell death. As the NP concentrations increased, the fluorescence in the treated samples decreased.

These results are consistent with the previously published studies in which ZnO NPs resulted in an increase in cytotoxicity in *Allium cepa* root cells [1]. In maize, a marked increase (∼1.8-fold) in Evans blue uptake was observed in roots exposed to ZnO NPs (<50 nm) as compared to the control [52]. The cytotoxic effect of ZnO NPs on marine algae with the increasing concentration (10–300 mg/L) showed decrease by 90% at 24 hours upon 50 mg/L ZnO NPs, whereas upon 300 mg/L, the viability of root cells significantly reduced up to 23% [69]. A similar appearance reported in the present study indicated that ZnO NPs-caused cytotoxicity was concentration- and time-dependent.

As ZnO NPs are absorbed by plants and translocated in the plant organism [70], it can be concluded that NPs induce cytotoxicity in seedlings, which can be related with mitotic inhibition or chromosomal aberrations. In previous investigations, the cytotoxic potential of ZnO NPs was found to be concentration-dependent, which is linked to mitotic inhibition. In addition, a previous analysis of chromosome morphology found a direct relationship between the increase in the number of aberrations and the increase in the concentration of NPs [71].

### 3.5. Stress Level

The generation of reactive oxygen species (ROS) in plants is part of the normal metabolism of chloroplasts, mitochondria, and peroxisomes and is one of the most common effects of stress, such as that caused by a toxic concentration of metal or metal NP exposure. The production of ROS in plants is necessary for normal plant growth and development; for example, it promotes cell proliferation and differentiation, secondary cell wall modification, and plant secondary metabolite production [72, 73]. In case of abiotic stress in plants, ROS can play several important roles. The most relevant role of ROS is the role in signal transduction reactions to mediate the activation of acclimation pathways, which has been found to prime plant defences to abiotic stress. In addition, ROS production in the chloroplast can avert electrons from the photosynthetic apparatus, preventing the overload of the antenna and subsequent damage. A similar function of ROS occurs in the mitochondria, where ROS production diverts electrons and thereby prevents the overload of different systems in the cell under stress. This function is possible because plant cells contain multiple levels of ROS detoxification pathways and mechanisms. Reactive oxygen species can also mediate the regulation of metabolic fluxes upon stress to prevent damage or overaccumulation of specific intermediates that are toxic to cells [72–76]. However, enhanced production of ROS can result in oxidative stress and affect metabolism via oxidative cell damage. Under evolutionary pressure, plants develop and expand a range of enzymatic and nonenzymatic ROS scavengers, such as antioxidant defence systems, which combine enzymatic and nonenzymatic antioxidants. Effective antioxidative systems in the symplastic compartments keep ROS concentrations low even under increased ROS production rates and thus achieve redox homeostasis. Homeostasis has a pivotal role in facilitating the toxicity of ROS and permitting normal cell growth [73, 77–80].

ROS function upon abiotic stress has also a negative side, such as their possible toxicity and the energetic costs related with their detoxification. Pathways require energy, and once this energy is exhausted, these pathways are unable to prevent ROS toxicity. Therefore, higher NP concentrations are highly toxic and cause an oxidative burst in plants; as a result, a reduction in antioxidant enzyme activity occurs in plants. Moreover, toxic amounts of ROS induce DNA, RNA, protein, and membrane oxidation and damage [33, 72, 73].

**Figure 4:**
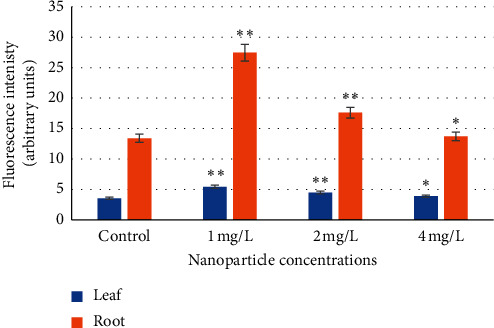
The effect of ZnO NPs on ROS generation in *H. vulgare* L. seedlings was studied using the fluorescent dye DCFDA. Values are the mean of three replicates with SD. ^*∗*^Significant difference from control (*p* < 0.01); ^*∗∗*^significant difference from control (*p* < 0.01).

According to the literature, ZnO NPs induce ROS production and reduce photosynthetic effectiveness and antioxidant activity in the wheat plant [4]. In Chinese mustard plants, ROS generation and the activities of antioxidant enzymes increased in response to the exposure to ZnO NPs at 200, 500, 1,000, and 1,500 *µ*g/mL [55]. These NPs can also reduce the oxidative stress in *Leucaena leucocephala* seedlings [64]. Moreover, antioxidant enzyme activities were increased and oxidative stress was decreased in wheat leaves exposed to the treatment of ZnO NPs at 100 ppm [32]. Evaluation of the effects of 10 nm ZnO NPs at concentrations of 500–4,000 mg/L on velvet mesquite in hydroponics demonstrated that these NPs generated increased catalase and peroxidase activity; however, the plants were visually healthy. These results suggest that these plants present a certain level of tolerance to ZnO NPs [71]. In the present study, antioxidant enzymes were not studied, but the results revealed increased oxidative stress in barley seedlings exposed to different concentrations of ZnO NPs. A significant (*p* < 0.01 and *p* < 0.05) increase in dichlorofluorescein (DCF) fluorescence intensity was observed for ZnO NP treatment with concentrations of 1, 2, and 4 mg/L (Figure 4), which reflects intracellular ROS production. The seedling length significantly increased in plants grown under NP stress. As plants activate their antioxidant systems and the level of oxidative stress decreases under increased ROS generation, it is possible that NPs in plant growing water solution are present all the time and can generate repeatedly increased ROS production and again plants are fighting with this stress. According to previous investigations, the oscillatory levels of ROS are related with Ca^2+^ gradients and pH fluctuations, which results in positive feedback to modulate polar growth over time [79]. It is possible that in the present study, the fluctuating ROS level in barley seedlings under NP stress led to an increase in seedling length.

The present study also observed more intensive DCF fluorescence in barley seedling roots than in leaves (Figure 4). This finding can be explained with several facts. The distinctive responses of antioxidative enzymes to ZnO in roots and leaves may refer to the varied levels of ROS generation, either by direct transfer of electrons in single-electron reactions involving metal cations, or as a consequence of metal-inactivated metabolic reactions. The accumulation of zinc oxide in leaves is lower than in roots, because the roots come in direct contact with engineered nanoparticles. Transport barriers also play a crucial role in NP translocation. Moreover, the oxidative damage inflicted by ZnO-engineered NPs is avoided with an increase in the activities of antioxidative enzymes [55]. The results of previous investigations of Chinese mustard *Brassica juncea* L. suggest that a ZnO NPs-induced enlargement in the levels of antioxidative enzymes can depict a secondary defensive mechanism against oxidative stress that is not as direct as primary defensive responses [55]. Moreover, the NP-mediated increase or decrease in antioxidant enzyme activities may be due to differences in plants, the type and size of NPs, and the exposure duration, under experimental conditions. The abovementioned studies indicate that plants can tolerate lower concentrations of NPs by increasing the action of antioxidants that scavenge ROS and consequently reach the balance between ROS formation and detoxification [33].

### 3.6. Genotoxicity and Genomic Template Stability

Genotoxicity describes the properties of chemical agents that damage the genetic information within a cell, causing mutations induced by NPs in plants [81]. Randomly amplified polymorphic DNA analysis is a rapid and reliable tool to monitor NP-induced biological effects in plants, as it is a sensitive method capable of detecting variations in genome profiles [68, 81, 82]. Five decamer primers were used to study the genotoxic effects of ZnO NPs in *H. vulgare* L. seedlings. All utilized primers generated a stable RAPD banding pattern. The results revealed differences between NP-treated and untreated plants, with clear variation in the number of amplified DNA bands for each primer. The polymorphism noticed in the RAPD profile involved the appearance of a new band (*a*) and the loss of a normal band (*b*) in comparison to the control RAPD profile. Band changes were detected in all experimental groups (Table 3). The number of total bands varied from three (OPA-11) to 15 (OPD-18). The largest average number of polymorphic bands per experimental group appeared in treated plants with NP concentrations of 4 mg/L; however, at concentrations of 1 mg/L and 2 mg/L, the numbers were lower: 2.2 and 2, respectively. Overall, the RAPD results demonstrate that ZnO NPs changed the genome of the barley seedlings. Moreover, many bands disappeared in the highest concentration of ZnO NPs compared to the control samples. This result can be explained by the highest concentration of ZnO NPs acting as a genotoxic agent causing DNA damage in the priming sites [1, 81].

**Figure 5:**
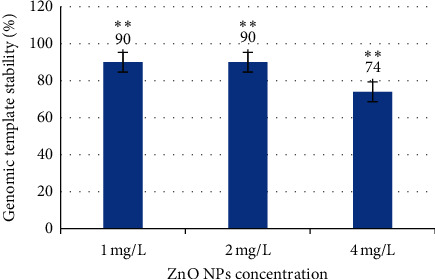
Comparison of genomic stability in seedlings of barley (*H. vulgare* L.) exposed to different concentrations of ZnO nanoparticles. Values are the mean of three replicates with SD. ^*∗∗*^Significant difference from control (*p* < 0.01).

Random amplified polymorphic DNA analysis was used to study the genomic stability in *H. vulgare* L. seedling leaves exposed to ZnO NPs. The GTS was calculated for all treated plants (Figure 5). The GTS for untreated seedlings was defined as 100%. There was a significant (*p* < 0.01) decrease in the GTS of all treated plant groups. The genome stability decreased by 10% in plants treated with 1 mg/L and 2 mg/L of NPs and by 26% in plants exposed to 4 mg/L of NPs. This result indicates that the most significant genome changes were induced by the higher NP concentration. Therefore, the results indicate that the ZnO NPs significantly reduce the stability of the barley seedling genome. From these results, DNA damage induced by ZnO NPs can be positively correlated with ROS generation.

Studies on genotoxicity caused by NPs, including ZnO NPs, in crucial crops have been widely reported in recent years. In such studies, the interaction of plant cells with ZnO NPs caused genotoxicity in plants, which is related with the modification of plant gene expression. However, ZnO NPs did not exhibit toxicity to cucumber plants and in organic rich soil at the concentration tested [63]. Moreover, ZnO NPs were found to be more toxic in solution culture than in soil culture [83]. However, DNA fragmentation and significant toxicity was also observed due to ZnO NPs <100 nm in size at concentrations of 0.4 g/L and 0.8 g/L in *Allium cepa* [1]. According to the literature, ZnO NPs can disturb cell division and cause mitotic aberrations, chromosomal breaks, and cell disintegration in the root tips of several plants [33, 84–86]. There are two pathways of genotoxicity: the direct and indirect pathway. The indirect pathway involves the reaction of NPs with mitochondria, which induces ROS generation; ROS generation, in turn, induces indirect DNA damage. In the direct pathway, NPs can cross the nuclear pore and interact with DNA, centromere, centrioles, and histone proteins through direct chemical or physical interaction [87]. In a previous study on fava beans and cultivated tobacco, the amount of ROS increased with ZnO NPs concentrations and ROS reacted with genomic DNA, leading to DNA strand break or damage [1]. Taken together, these results indicate that the generation of ROS by plants under NP stress can lead to DNA damage and protein oxidation, among other effects [72], which can result in RAPD profile changes and thereby changes in GTS. Most previous studies were conducted *in vitro*; therefore, it may be valuable to perform investigations *in vivo* to understand the mechanisms behind the genotoxic effects of NPs in crop plants [33].

### 3.7. MicroRNA Expression Levels

**Figure 6:**
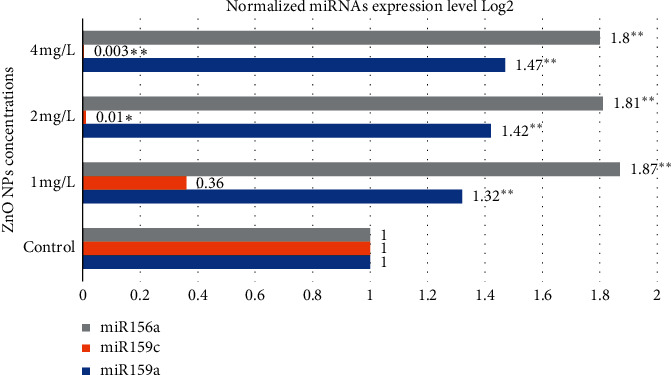
The results of the determination of miR156a, miR159a, and miR159c expression levels in control and experimental groups of barley (*H. vulgare* L.) plants exposed to different concentrations of ZnO nanoparticles. Values are the mean of three replicates with SD. ^*∗*^Significant difference from control (*p* < 0.05); ^*∗∗*^significant difference from control (*p* < 0.01).

The quantitative real-time polymerase chain reaction (qRT-PCR) is a technique to quantify gene expression [88, 89]. In the present study, the qRT-PCR method was used to quantify the miRNA expression level. The results demonstrate that ZnO NP treatment altered the expression of all miRNAs (miR156a, miR159a, and miR159c) in a dosage-dependent manner (Figure 6). The miR156a and miR159a expression levels were significantly (*p* < 0.01) upregulated at all tested concentrations; however, 2 mg/L and 4 mg/L NP exposure significantly (*p* < 0.05 and *p* < 0.01, resp.) downregulated miR159c expression when compared to the controls. The expression level of miR159c declined with increasing NP concentrations.

Generally, microRNAs act as negative regulators of gene expression in eukaryotes [41, 42]; however, some miRNAs can promote the expression of target genes by downregulating themselves [90]. Moreover, miRNAs are involved in many kinds of abiotic and biotic stress responses in plants. The same miRNA can be involved in different response mechanisms in different plant species [45, 46]. There are very few scientific articles which have examined the effect of engineered ZnO NPs on miRNA expression in plants. Adhikari et al. [52] demonstrated the effect of ZnO NPs on miRNA expression in maize. Their study revealed considerable downregulation of miR156a and miR159a expression in maize plants under ZnO NP treatment. Nevertheless, the results of the present study indicate an opposite effect: the expression of these two miRNAs in barley seedlings was significantly upregulated under ZnO NP treatment. These miRNAs are involved in various plant responses to stress, such as responses to drought, hypoxia, fungal infections, and NPs [47, 48]. For example, the expression of miR156 is repressed in wheat plants in response to powdery mildew infection. In addition, miR156 is downregulated under fungal infection in pine and wheat, and miR159 is downregulated in response to fungi in wheat [49, 91]. Researchers have found that miR156 and miR159 in maize are involved in plant growth and development. In addition, miR156 is associated with ROS homeostasis in maize [52]. Due to the complexity of miRNA regulation mechanisms and the large variety of miRNAs and plant species, comprehension of the regulating mechanism of action of miRNAs is still limited [42, 52]. As miR156 and miR159 are downregulated in response to fungal infection and the present study found upregulation of these miRNAs in barley under ZnO NP treatment, it is necessary to investigate the effect of ZnO NPs in barley infected with powdery mild pathogens. The presence of these NPs may potentially increase the expression of miR156 and miR159, which would enhance barley resistance to fungal pathogens.

## 4. Conclusions

The present study is one of the first focused, systematic studies to highlight the effect of ZnO NPs on barley *H. vulgare* L. seedlings. The results revealed that ZnO NPs (32 nm) at concentrations of 1, 2, and 4 mg/L enhanced barley seed germination, shoot/root elongation, and H_2_O_2_ stress level and decreased root cell viability and genomic template stability. Moreover, the NPs up- and downregulated miR156a, miR159a, and miR159c. However, less is known about the effect of ZnO NPs on crop plant growth, development, and stress response mechanisms. Therefore, further comprehensive field studies are needed to understand the impact mechanisms of ZnO NPs in crop plants and to inform the practical application of ZnO NPs to increase barley resistance to fungal pathogens.

## Figures and Tables

**Table 1:** The germination percentage of seeds, shoot length, root length, and number of seminal roots in *H. vulgare* L. seedlings grown 7 days with 0, 1, 2, and 4 mg/L of ZnO NPs.

	Germination (%)	Shoot length (mm)	Root length (mm)	Seminal roots (*n*)
Control	42 ± 1.57	36.8 ± 2.09	28.2 ± 0.91	6.8 ± 1.1
1 mg/L	63 ± 1.82^*∗∗*^	49.3 ± 2.39^*∗*^	36.4 ± 0.89	7.2 ± 0.84
2 mg/L	57 ± 1.76^*∗∗*^	46.5 ± 1.67^*∗*^	33.6 ± 0.67	7.6 ± 0.55
4 mg/L	66 ± 1.89^*∗∗*^	52.4 ± 1.53^*∗∗*^	43.4 ± 1.48	7.8 ± 0.84

Values are the mean of three replicates with SD. ^*∗*^Significant difference from control (*p* < 0.05); ^*∗∗*^significant difference from control (*p* < 0.01).

**Table 2:** Shoot length, root length, number of seminal roots, and shoot weight in *H. vulgare* L. seedlings grown 14 days with 0, 1, 2, and 4 mg/L of ZnO NPs.

	Shoot length (cm)	Root length (cm)	Number of roots	Shoot weight (mg)
Control	16.19 ± 1.73	3.95 ± 1.37	10.75 ± 1.04	15.96 ± 2.51
1 mg/L	21 ± 2.39^*∗∗*^	6.21 ± 0.99^*∗∗*^	9.25 ± 1.17^*∗*^	17.11 ± 1.47
2 mg/L	20.63 ± 2.00^*∗∗*^	7.44 ± 1.32^*∗∗*^	8.88 ± 0.83^*∗∗*^	17.11 ± 3.77
4 mg/L	20.63 ± 1.69^*∗∗*^	7.06 ± 1.55^*∗∗*^	8.25 ± 1.16^*∗∗*^	15.86 ± 2.20

Values are the mean of three replicates with SD. ^*∗*^Significant difference from control (*p* < 0.05); ^*∗∗*^significant difference from control (*p* < 0.01).

**Table 3:** The results of *H. vulgare* L. seedling RAPD analysis.

Primer ID	Primer sequences (5′-3′)	Length (bp)	Number of polymorphic bands of 1 mg/L		Number of polymorphic bands of 2 mg/L		Number of polymorphic bands of 4 mg/L		Total number of bands
			*a*	*b*	*a*	*b*	*a*	*b*	
CB-21	CAGCACTGAC	10	3	3	2	3	3	3	9
OPA-02	TGCCGAGCTG	10	3	2	0	1	4	5	11
OPA-05	AGGGGTCTTG	10	0	0	1	1	1	1	4
OPA-11	CAATCGCCGT	10	0	0	0	0	0	1	3
OPD-18	GAGAGCCAAC	10	0	0	0	2	7	5	15
Total polymorphic bands			6	5	3	7	15	15	
Average number of polymorphic bands per experimental group			2.2		2		6		

Primers used, number of polymorphic bands in plants treated with 1 mg/L, 2 mg/L, and 4 mg/L of ZnO NPs, total number of bands for each primer, and average number of polymorphic bands for every plant group. *a*: new band; *b*: disappeared band.

## Data Availability

The data used to support the findings of this study are included within the article.
